# Multimodal Assessment of Biological Age Following Radiation Therapy Among Patients With Early-Stage NSCLC

**DOI:** 10.1001/jamanetworkopen.2026.4872

**Published:** 2026-04-08

**Authors:** Grace Lee, Fridolin Haugg, Dennis Bontempi, John He, Danielle S. Bitterman, Suraj Pai, Christian Guthier, Kelly J. Fitzgerald, David E. Kozono, Benjamin H. Kann, Hugo J. W. L. Aerts, Raymond H. Mak

**Affiliations:** 1 Department of Radiation Oncology, Mass General Brigham/Dana-Farber Cancer Institute, Harvard Medical School, Boston, Massachusetts; 2 Artificial Intelligence in Medicine (AIM) Program, Mass General Brigham, Harvard Medical School, Boston, Massachusetts; 3 Department of Radiology and Nuclear Medicine, CARIM & GROW, Maastricht University, Maastricht, the Netherlands; 4 Department of Radiation Oncology (MAASTRO), Maastricht University, Maastricht, the Netherlands; 5 Department of Radiology, Brigham and Women’s Hospital, Harvard Medical School, Boston, Massachusetts

## Abstract

**Importance:**

Accurately estimating physiological fitness and life expectancy is challenging in older patients with early-stage non–small cell lung cancer (NSCLC) considered for stereotactic body radiotherapy (SBRT). Noninvasive biological age metrics may improve assessment beyond chronological age.

**Objective:**

To evaluate whether a photography-based age estimating algorithm (face age) and spirometry-based estimated age (lung age) are associated with overall survival and early mortality.

**Design, Setting, and Participants:**

This retrospective cohort study included patients aged 60 years and older with early-stage NSCLC treated with definitive SBRT from June 2009 to March 2023. Participants were recruited from 6 radiation oncology clinics affiliated with a single academic cancer center and had a median follow-up of 44 months. Pretreatment identification photographs were available for face age estimation in all patients; baseline pulmonary function tests were available for a subset and used to compute lung age.

**Exposures:**

Face age used a validated deep learning model to estimate age from pre-SBRT photographs; lung age was derived from spirometry using validated equations.

**Main Outcomes and Measures:**

Primary outcomes were overall survival and 2-year mortality. Multivariate Cox models adjusted for clinical covariates.

**Results:**

Among 670 patients (median [range] age, 77 [60-98] years; 406 [61%] female), the median (range) face age was 79 (range, 47-98) years. Overall, 113 participants (17%) vs 149 (22%) were 85 years or older by chronological age vs face age, respectively. The median overall survival was 47 (95% CI, 42-54) months, and 161 (24%) died within 2 years. On multivariate analysis adjusting for sex, Eastern Cooperative Oncology Group performance status, cancer stage, smoking pack-years, and histology, older face age was associated with worse overall survival (adjusted hazard ratio [HR] per decade, 1.39; 95% CI, 1.13-1.71; *P* = .002), whereas chronological age was not. Older face age and face age of 85 years or older were associated with increased 2-year mortality (adjusted HR per decade, 1.35; 95% CI, 1.03-1.78; *P* = .03; age ≥85 years: adjusted HR, 1.59; 95% CI, 1.06-2.40; *P* = .03), whereas chronological age per decade and age of 85 years or older were not. In the subset of 477 participants with spirometry data, median lung age was 98 (range, 22-139) years and showed minimal correlation with face age (*r* = 0.07). Face age remained independently associated with survival in sensitivity analyses adjusting for lung age.

**Conclusions and Relevance:**

In this cohort study of older patients with early-stage NSCLC receiving SBRT, face age was an independent biomarker associated with survival and early mortality and complemented lung age. These noninvasive measures, obtainable from routine photographs and spirometry, may aid individualized risk stratification and treatment planning.

## Key Points

**Question:**

Are photography-based (face age) and spirometry-based (lung age) age estimates relevant to outcomes beyond chronological age in older patients with early-stage non–small cell lung cancer (NSCLC) treated with stereotactic body radiotherapy (SBRT)?

**Findings:**

In this cohort study of 670 patients aged 60 years or older, face age was significantly associated with overall survival and 2-year mortality, whereas chronological age was not independently associated. Lung age showed minimal correlation with face age, suggesting that these biomarkers capture distinct physiological domains.

**Meaning:**

The findings of this study suggest that easily obtained biological age measures from routine photographs and spirometry may help personalize SBRT decision-making for older patients with early-stage NSCLC.

## Teaser

This cohort studies evaluates whether photography-based and spirometry-based age estimates are associated with overall and 2-year survival among adults aged 60 years and older with non–small cell lung cancer (NSCLC) who received definitive stereotactic body radiotherapy.

## Introduction

Lung cancer is the second most common cancer and the leading cause of cancer-related death worldwide, accounting for 11% of new diagnoses and 18% of all cancer mortalities.^1^ Non–small cell lung cancer (NSCLC) constitutes 80% to 85% of all lung cancers.^2^ In the United States, 27% of lung cancers are diagnosed at a localized stage.^3^ Although surgical resection is the primary treatment for early-stage NSCLC, many patients are deemed inoperable due to comorbid conditions, such as pulmonary or cardiovascular disease.^4^ For these patients, stereotactic body radiation therapy (SBRT) is a safe and effective definitive treatment.^5,6^ Its use is growing even in operable cases, but patients who receive SBRT instead of surgery tend to be older and have significant comorbidities.^7^ This growing reliance on SBRT, particularly among older, high-risk patients, underscores the urgent need for accurate assessments of biological fitness and prognoses.^8^

Assessment of frailty and life expectancy in such a high-risk patient cohort is crucial for making well-informed, personalized treatment decisions.^9^ Patients with limited life expectancy may derive minimal benefit from definitive treatment and could be considered for observation, whereas biologically younger patients may be optimal candidates for curative-intent SBRT. Despite its importance, precise prognostication poses a challenging task even for experienced clinicians.^10,11,12,13^ Recent advances in artificial intelligence (AI) offer promising approaches to quantify biological aging, with deep learning models showing superior discriminative ability compared with traditional clinical parameters in NSCLC.^14^ While age is strongly associated with mortality, biological age markers may better reflect an individual’s health and fitness.^15^ Various age biomarkers have been explored, including imaging-based quantification^16,17^; molecular data, such as epigenomic clocks^18^; and physiological measures, such as pulmonary age estimation from lung function tests.^19^

We previously developed a deep learning model that estimates biological age from facial photographs and demonstrated its association with survival outcomes in a general cancer population.^20^ A complementary biological age metric derived from pulmonary function tests (PFT) quantifies respiratory aging based on forced expiratory volume in one second (FEV_1_) and has been independently associated with mortality and cardiovascular outcomes, even in individuals without clinically significant impairment.^21,22^ Baseline pulmonary function is routinely used to assess candidacy for lung-directed local therapy such as surgery and radiation.

The current study evaluates the photography-estimated age model (face age) as a biological aging marker in patients with early-stage NSCLC undergoing SBRT, assessing its association with outcomes in this high-risk population. Additionally, we explore whether spirometry-estimated age (lung age) captures complementary physiological information independent of face age.

## Methods

This study was conducted in accordance with the Strengthening the Reporting of Observational Studies in Epidemiology (STROBE) reporting guideline for cohort studies and the Transparent Reporting of a Multivariate Prediction Model for Individual Prognosis or Diagnosis for Artificial Intelligence (TRIPOD-AI)^23^ guidelines for reporting clinical prediction models incorporating artificial intelligence. This retrospective cohort study was approved by the Dana Farber/Harvard Cancer Center institutional review board, with a waiver of informed consent because this was a retrospective study of existing routine care clinical records and photographs, posed minimal risk, involved no participant contact, and included many deceased individuals.

### Study Design and Patient Selection

Patients aged 60 years and older who underwent definitive stereotactic body radiotherapy (SBRT) for early-stage (*American Joint Commission on Cancer* 8th edition stages T1 to 3N0M0, stage I-II) NSCLC with or without biopsy confirmation between June 2009 and March 2023 at 6 clinics were identified. To be included in the study, patients had to have a pre-SBRT facial photograph used for identification as well as post-SBRT follow-up data available. Clinical variables collected from the electronic health record included chronological age, sex, race or ethnicity, Eastern Cooperative Oncology Group performance status (ECOG PS), smoking history, tumor size, TNM stage, histology, body mass index (BMI; calculated as weight in kilograms divided by height in meters squared), and PFTs, including FEV_1_ and diffusing capacity for carbon monoxide (Dlco) obtained within 2 years prior to SBRT, if available. Race and ethnicity were self-reported and collected as part of routine electronic health record documentation and used here to describe the cohort and assess potential differences in model performance across groups. The patient selection process is illustrated in eFigure 1 in Supplement 1.

### Face Age

FaceAge is a deep learning model that was trained on facial images of more than 56 000 healthy individuals to estimate biological age from a single face photograph; in prior validation datasets, it achieved a mean absolute error of 4.09 years for individuals aged 60 years and older.^20^ The study cohort is fully independent from the model training data. Each patient’s pre-SBRT identification photograph was processed by the algorithm, producing a face age. The difference between face age and chronological age was then computed to characterize the relative concordance or discordance of aging.

### Lung Age

Among patients with baseline PFT data, lung age was calculated from height and observed FEV_1_ using validated equations.^19^ For men, lung age = (2.87 × height in inches) – (31.25 × FEV_1_ in liters) – 39.375; for women, lung age = (3.56 × height in inches) – (40.00 × FEV_1_ in liters) – 77.28.

### Mean Age Estimation

In select analyses, the mean of face and lung age was taken to generate a combined, multimodality biological age metric. We also explored a standardized approach using *z*-score normalization to address potential scale discrepancies between the metrics. This alternative method transformed both estimated ages to have mean of 0 and SD of 1 before averaging them.

### End Points

Primary end points were overall survival (OS) and early mortality, defined as death within 2 years. Date of death was obtained from medical records. Secondary end points were distant metastasis and symptomatic (Common Terminology Criteria for Adverse Events [CTCAE] grade ≥2) radiation pneumonitis. All end points were measured from the date of the pre-SBRT identification photograph.

### Median Follow-Up

Patients were typically followed up with chest computed tomography (CT) scans and radiation oncologist visits every 6 months for the first 2 years, then annually. Median follow-up time was calculated using the reverse Kaplan-Meier method, which estimates potential follow-up by treating death as a censoring event and censoring as the event of interest.

### Statistical Analysis

Descriptive statistics summarized patient demographic characteristics, disease characteristics, and distributions of age metrics (chronological age, face age, and lung age). Cox proportional hazards models were used to analyze OS, 2-year mortality, and distant metastasis; logistic regression was used for grade 2 or greater pneumonitis. Time dependent area under the receiver operating characteristic (ROC) curve (AUC) at 1 and 2 years were estimated using a time dependent ROC approach, with 95% CIs obtained by bootstrap resampling. For the 2-year mortality end point, Cox regression with administrative censoring at 24 months was used to include all patients and properly account for those with shorter follow-up, avoiding the selection bias that would result from excluding patients still alive with less than 2 years of follow-up. In multivariate models, chronological age and face age metrics of interest (continuous or thresholded) were incorporated along with clinical covariates showing *P* < .10 in univariate analyses. Face age of 85 years or older was used as the cutoff to define very elderly patients, as prior evidence indicates significantly worse survival and increased toxic effects for individuals aged 85 years or older receiving SBRT for early-stage NSCLC.^8^ Additional categorical variables (difference between age and face age ≥10 years and ≤−5 years) were created to identify patients with substantial discordance between biological and chronological age, focusing on extreme cases in which patients appeared much older or younger than their chronological age. Clinical covariates incorporated included sex, ECOG PS, smoking pack years, stage, and histology. PFT metrics (FEV_1_, Dlco, lung age) were included in sensitivity analyses of the subgroup with available data. Kaplan-Meier estimates of survival were compared using log-rank tests. A 2-sided *P* < .05 was considered statistically significant. All analyses were conducted using R version 4.3.1 (R Project for Statistical Computing).

## Results

### Patient Characteristics

A total of 670 patients meeting the study criteria were identified. Baseline characteristics are summarized in Table 1. The median (range) age was 77 (60-98) years, and 406 (61%) were female. Overall, 21 participants (3%) were Black, 4 (1%) were Asian, and 635 (95%) were White; 142 (21%) had an ECOG PS of 0, and 526 (79%) reported active smoking. The median (range) tumor size was 1.7 (0.6-6.0) cm. Stage IA disease (IA1-3) accounted for 90% of tumors (604 participants), with the remaining 10% being IB or IIA/B. PFT data were available in a subset of patients, with 479 (73%) having an FEV_1_ measurement, and 266 (53%) having a Dlco. The median (range) FEV_1_ was 1.4 (0.3-3.9) L. The median (range) predicted percentages were 65% (20%-175%) for FEV_1_ and 49% (9%-112%) for Dlco.

**Table 1.** Patient Characteristics

Variables	Patients, No. (%) (N = 670)
Age, median (range), y	76.8 (60.2 to 97.7)
Follow-up, median (range), mo	44.1 (0.2 to 171.5)
Face age	
Median (range), y	79.3 (46.7 to 97.5)
≥85 y	149 (22)
Difference from chronological age, y	
Median (range)	2.2 (−29.7 to 22.3)
≥10	81 (12)
≥5	222 (33)
≤−5	98 (15)
Sex	
Female	406 (61)
Male	264 (39)
Race and ethnicity	
Black	21 (3)
Asian	4 (1)
White	635 (95)
Other^a^	4 (1)
Unknown	6
ECOG PS	
0	142 (21)
1	328 (49)
2	165 (25)
3	34 (5)
Unknown	1
Smoking	
Never	42 (6)
Former	102 (15)
Active	526 (79)
Smoking pack years, median (range) (n = 626)	45 (0 to 240)
Tumor size, median (range), cm	1.7 (0.6 to 6.0)
Stage	
IA1	94 (14)
IA2	354 (53)
IA3	156 (23)
IB	47 (7)
IIA	13 (2)
IIB	6 (1)
Multifocal	280 (42)
Histology	
Adenocarcinoma	203 (30)
Squamous cell carcinoma	99 (15)
Other or NOS	30 (4)
Unknown (not biopsied)	338 (50)
BMI, median (range)	26.3 (13.7 to 52.7)
PFT values, median (range)	
FEV_1_, L (n = 479)	1.4 (0.3 to 3.9)
FEV_1_, % predicted (n = 486)	65.0 (20.0 to 175.0)
Dlco, mL/min/mm Hg (n = 266)	10.1 (2.1 to 23.9)
Dlco, % predicted (n = 356)	49.0 (9.0 to 112.0)
Lung age, median (range) (n = 477)	97.5 (21.5 to 139.0)
Mean estimated age, median (range) (n = 477)	87.6 (47.5 to 111.0)
Normalized mean estimated age, median (range) (n = 477)	0.02 (−2.65 to 1.50)

Abbreviations: BMI, body mass index (calculated as weight in kilograms divided by height in meters squared); Dlco, diffusing capacity for carbon monoxide; ECOG PS, Eastern Cooperative Oncology Group performance; FEV_1_, forced expiratory volume in 1 second; NOS, not otherwise specified; PFT, pulmonary function test.

^a^ Patients self-reported other race and ethnicity.

### Face and Lung Age Distributions and Correlation

**Figure 1.**
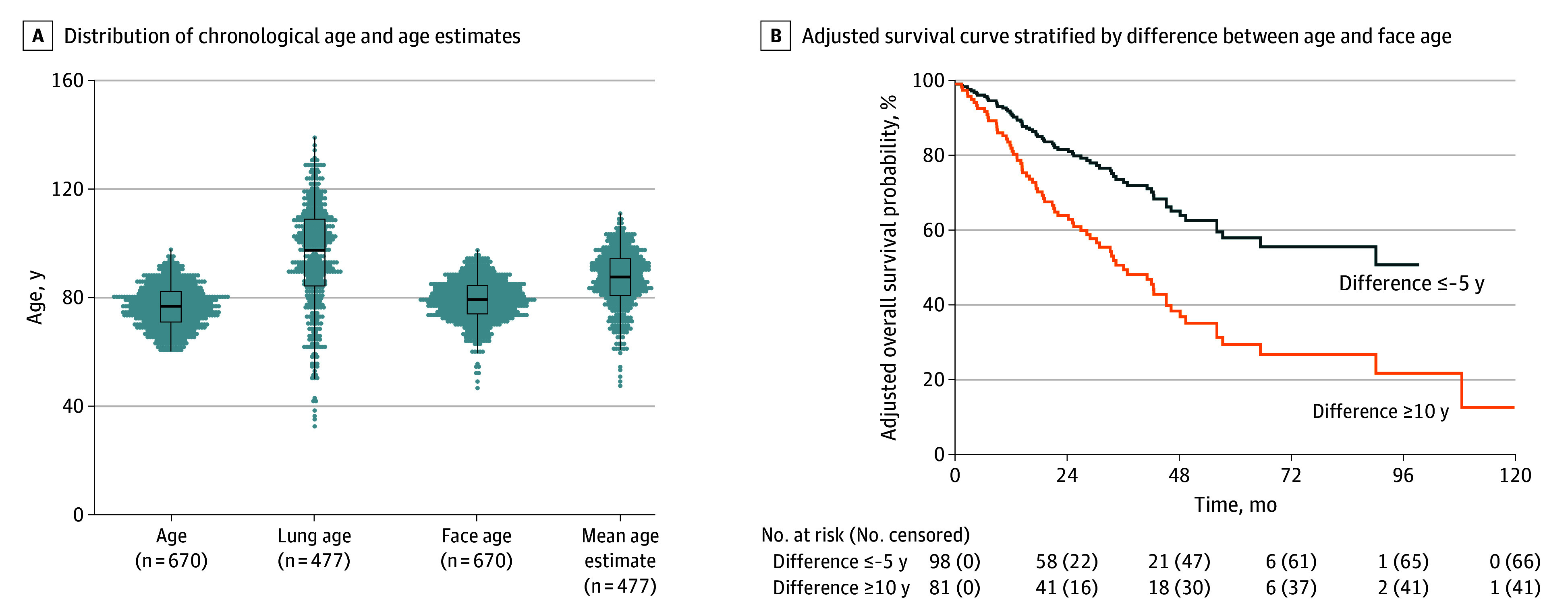
Box Plots of Age Biomarker Distributions and Survival Curve Based on Difference Between Face Age and Chronological Age A, Distribution of age, lung age, face age, and mean estimated age. Center line shows the median and box bounds the IQR, whiskers extend to 1.5 × IQR, and dots show individual observations stacked horizontally to indicate distribution density. B, Adjusted survival curve stratified by difference between age and face age, stratified into 2 groups based on the difference: −5 years or less and 10 years or more. Survival curves were adjusted for chronological age using Cox proportional hazards regression.

Among the entire cohort, the median (range) face age was 79 (47-98) years. Face age exceeded chronological age in 427 patients (64%), with a median (range) difference between face age and age of 2.2 (−29.7 to 22.3) years. The median (range) lung age, calculated for 477 patients with baseline FEV_1_ and height data, was 98 (22 to 139) years. Distributions of chronological age, face age, and lung age are shown in Figure 1A, and associations among these aging metrics are illustrated in eFigure 2 in Supplement 1. Lung age exhibited the widest range and was only weakly correlated with either chronological age (*r* = 0.07) or face age (*r* = 0.07), suggesting it captures a distinct aspect of physiological aging. In contrast, face age showed a moderate correlation with chronological age (*r* = 0.60).

### OS

There was a median (range) follow-up of 44 (0-172) for all patients and 31 (1-172) months for surviving patients. The median OS was 47 (95% CI, 42-54) months.

**Figure 2.**
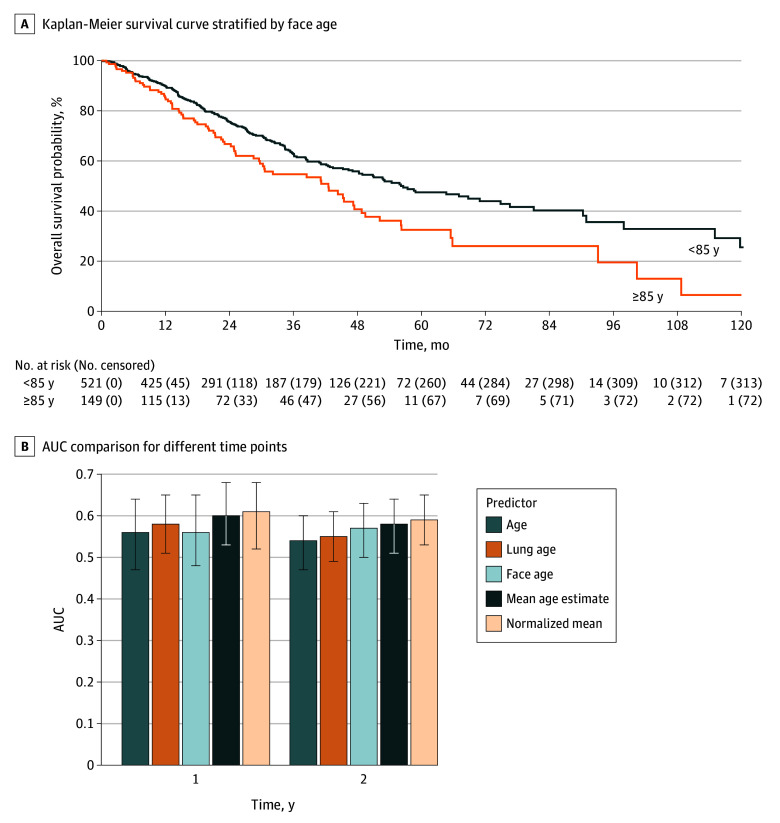
Survival Curve Stratified by Face Age and Bar Graph of Discrimination of Age Metrics A, Kaplan-Meier survival curve stratified by face age. Patients were categorized into 2 groups based on their face age: younger than 85 years and 85 years and older. B, Time dependent area under the curve (AUC) at 1 and 2 years for chronological age, face age, lung age, and combined multimodal metrics. Error bars indicate 95% confidence intervals estimated using 1000 bootstrap resamples.

On multivariate Cox analysis (Table 2), older face age was significantly associated with worse OS (adjusted hazard ratio [HR] per decade, 1.39; 95% CI, 1.13-1.71; *P* = .002), whereas chronological age was not after adjusting for other clinical factors (sex, ECOG PS, smoking pack-years, stage, and histology). Patients with face age of 85 years or older had worse OS (adjusted HR, 1.42; 95% CI, 1.05-1.93; *P* = .02). Patients with a large discordance between face age and chronological age (difference ≥10 years) had an adjusted HR of 1.45, but the finding was not statistically significant (95% CI; 1.00–2.12; *P* = .05). Adjusted survival curves in Figure 1B show that much older face age relative to chronological age was associated with significantly reduced survival (HR 2.25; 95% CI, 1.21-4.18; *P* = .01), while much younger face age than chronological age was associated with more favorable outcomes. Kaplan-Meier analysis of patients with face age of 85 years or older vs younger than 85 years is shown in Figure 2A (*P* = .003).

**Table 2.** Multivariate Cox Regression for Overall Survival

Variable	Multivariate model (face age)^a^		Multivariate model (face age *≥*85 y)^a^		Multivariate model (face age − age)^a^		Multivariate model (face age − age ≥10 y)^a^		Multivariate model (face age − age ≤−5 y)^a^	
	HR (95% CI)	*P* value	HR (95% CI)	*P* value	HR (95% CI)	*P* value	HR (95% CI)	*P* value	HR (95% CI)	*P* value
Chronological age (decade)	1.04 (0.85-1.27)	.69	1.15 (0.96-1.38)	.13	1.45 (1.20-1.75)	<.001	1.32 (1.11-1.56)	.002	1.32 (1.12-1.56)	.001
Face age										
Face age (decade)	1.39 (1.13-1.71)	.002	NA	NA	NA	NA	NA	NA	NA	NA
Face age *≥*85 y	NA	NA	1.42 (1.05-1.93)	.02	NA	NA	NA	NA	NA	NA
Face age − age (decade)	NA	NA	NA	NA	1.39 (1.13-1.71)	.002	NA	NA	NA	NA
Face age − age *≥*10 y	NA	NA	NA	NA	NA	NA	1.45 (1.00-2.12)	.05	NA	NA
Face age − age *≤*−5 y	NA	NA	NA	NA	NA	NA	NA	NA	0.65 (0.44-0.96)	.03
Sex, male vs female										
Female	1 [Reference]	NA	1 [Reference]	NA	1 [Reference]	NA	1 [Reference]	NA	1 [Reference]	NA
Male	1.65 (1.29-2.11)	<.001	1.58 (1.24-2.02)	<.001	1.65 (1.29-2.11)	<.001	1.62 (1.27-2.07)	<.001	1.59 (1.24-2.02)	<.001
ECOG PS										
0-1	1 [Reference]	NA	1 [Reference]	NA	1 [Reference]	NA	1 [Reference]	NA	1 [Reference]	NA
2-4	2.43 (1.90-3.10)	<.001	2.46 (1.92-3.14)	<.001	2.43 (1.90-3.10)	<.001	2.44 (1.91-3.12)	<.001	2.39 (1.87-3.05)	<.001
Smoking pack-years	1.00 (1.00-1.00)	.52	1.00 (1.00-1.00)	.56	1.00 (1.00-1.00)	.52	1.00 (1.00-1.00)	.50	1.00 (1.00-1.00)	.51
Stage										
I (IA1-IB)	1 [Reference]	NA	1 [Reference]	NA	1 [Reference]	NA	1 [Reference]	NA	1 [Reference]	NA
II (IIA-IIB)	1.73 (0.95-3.14)	.07	1.75 (0.96-3.19)	.07	1.73 (0.95-3.14)	.07	1.59 (0.88-2.88)	.13	1.63 (0.90-2.96)	.11
Histology										
Adenocarcinoma	1 [Reference]	NA	1 [Reference]	NA	1 [Reference]	NA	1 [Reference]	NA	1 [Reference]	NA
Squamous cell carcinoma	1.68 (1.18-2.38)	.004	1.78 (1.25-2.52)	.001	1.68 (1.18-2.38)	.004	1.73 (1.22-2.45)	.002	1.74 (1.23-2.46)	.002
Other or NOS	1.40 (0.87-2.26)	.16	1.44 (0.90-2.32)	.13	1.40 (0.87-2.26)	.16	1.41 (0.87-2.27)	.16	1.50 (0.93-2.41)	.10
Unknown (not biopsied)	1.03 (0.77-1.37)	.86	1.02 (0.77-1.37)	.87	1.03 (0.77-1.37)	.86	1.02 (0.77-1.37)	.88	1.05 (0.78-1.40)	.77

Abbreviations: ECOG PS, Eastern Cooperative Oncology Group performance; NA, not applicable; NOS, not otherwise specified.

^a^ Results for 626 participants with 283 events; tumor size excluded from multivariate model given confounding with stage; pulmonary function test; lung age, mean estimated age, and normalized mean estimated age variables excluded from multivariate model given data available for only a subset of patients.

In sensitivity analyses restricted to the subset with PFT data (477 had lung age calculated; 449 had complete data for multivariate analysis with lung age; 339 for Dlco analysis), face age remained significantly associated with OS even when lung age was added to the model (eTable 1 in Supplement 1). Notably, both lung age and Dlco were significantly associated with OS in univariate analysis (lung age: HR per decade, 1.11; 95% CI 1.03-1.18; *P* = .004; Dlco: HR 0.93; 95% CI 0.89-0.97, *P* < .001) (eTable 5 in Supplement 1). In the multivariate model including both aging metrics, face age maintained statistical significance (adjusted HR, 1.38; 95% CI, 1.09-1.75; *P* = .007) after adjusting for lung age (Table 2 and eTable 1 in Supplement 1).

In exploratory analyses, combined face age and lung age measures showed the highest discrimination among the evaluated metrics, with AUCs for mean estimated age of 0.60 (95% CI, 0.53-0.68) and 0.58 (95% CI, 0.51-0.64) at 1 and 2 years, respectively, and AUC for normalized mean estimated age of 0.61 (95% CI, 0.52-0.68) and 0.59 (95% CI, 0.53-0.65) (Figure 2B).

### Early Mortality

Among all 670 patients, 161 (24%) died within 2 years. In multivariate Cox regression with administrative censoring at 24 months (Table 3), older face age as a continuous variable was associated with higher 2-year mortality risk (adjusted HR per decade, 1.35; 95% CI 1.03-1.78; *P* = .03), while chronological age was not. Patients with face age of 85 years or older had increased risk of early mortality (adjusted HR, 1.59; 95% CI, 1.06-2.40; *P* = .03), whereas chronological age of 85 years or older was not significant. A substantial positive difference between face age and chronological age (≥10 years) was significantly associated with higher 2-year mortality (adjusted HR, 1.67; 95% CI, 1.02-2.75; *P* = .04). Univariate results and sensitivity analyses adjusting for lung age and Dlco are presented in eTables 2 and 5 in Supplement 1.

**Table 3.** Multivariate Cox Regression for 2-Year Mortality

Variable	Multivariate model (face age)^a^		Multivariate model (face age *≥*85 y)^a^		Multivariate model (face age − age)^a^		Multivariate model (face age − age ≥10 y)^a^		Multivariate model (face age − age ≤−5 y)^a^	
	HR (95% CI)	*P* value	HR (95% CI)	*P* value	HR (95% CI)	*P* value	HR (95% CI)	*P* value	HR (95% CI)	*P* value
Chronological age (decade)	0.93 (0.71-1.21)	.58	0.98 (0.76-1.25)	.85	1.25 (0.98-1.60)	.08	1.19 (0.94-1.50)	.14	1.15 (0.92-1.45)	.21
Face age										
Face age (decade)	1.35 (1.03-1.78)	.03	NA	NA	NA	NA	NA	NA	NA	NA
Face age *≥*85 y	NA	NA	1.59 (1.06-2.40)	.03	NA	NA	NA	NA	NA	NA
Face age − age (decade)	NA	NA	NA	NA	1.35 (1.03-1.78)	.03	NA	NA	NA	NA
Face age − age *≥*10 y	NA	NA	NA	NA	NA	NA	1.67 (1.02-2.75)	.04	NA	NA
Face age − age *≤*-5 y	NA	NA	NA	NA	NA	NA	NA	NA	0.67 (0.40-1.13)	.13
Sex										
Female	1 [Reference]	NA	1 [Reference]	NA	1 [Reference]	NA	1 [Reference]	NA	1 [Reference]	NA
Male	1.51 (1.09-2.10)	.01	1.47 (1.06-2.05)	.02	1.51 (1.09-2.10)	.01	1.53 (1.10-2.13)	.01	1.47 (1.06-2.05)	.02
ECOG PS										
0-1	1 [Reference]	NA	1 [Reference]	NA	1 [Reference]	NA	1 [Reference]	NA	1 [Reference]	NA
2-4	2.83 (2.03-3.93)	<.001	2.89 (2.08-4.03)	<.001	2.83 (2.03-3.93)	<.001	2.90 (2.08-4.03)	<.001	2.82 (2.02-3.92)	<.001
Smoking pack years	1.00 (1.00-1.01)	.44	1.00 (1.00-1.01)	.50	1.00 (1.00-1.01)	.44	1.00 (1.00-1.01)	.41	1.00 (1.00-1.01)	.49
Stage										
I (IA1-IB)	1 [Reference]	NA	1 [Reference]	NA	1 [Reference]	NA	1 [Reference]	NA	1 [Reference]	NA
II (IIA-IIB)	2.17 (1.11-4.24)	.02	2.27 (1.16-4.46)	.02	2.17 (1.11-4.24)	.02	2.02 (1.04-3.94)	.04	2.09 (1.07-4.07)	.03
Histology										
Adenocarcinoma	1 [Reference]	NA	1 [Reference]	NA	1 [Reference]	NA	1 [Reference]	NA	1 [Reference]	NA
Squamous cell carcinoma	1.55 (0.98-2.43)	.06	1.63 (1.04-2.56)	.04	1.55 (0.98-2.43)	.06	1.57 (1.00-2.47)	.049	1.59 (1.01-2.50)	.04
Other or NOS	0.76 (0.34-1.71)	.51	0.79 (0.35-1.77)	.57	0.76 (0.34-1.71)	.51	0.75 (0.33-1.69)	.49	0.81 (0.36-1.83)	.62
Unknown (not biopsied)	1.11 (0.75-1.64)	.62	1.11 (0.75-1.65)	.60	1.11 (0.75-1.64)	.62	1.11 (0.75-1.64)	.62	1.13 (0.76-1.68)	.55

Abbreviations: ECOG PS, Eastern Cooperative Oncology Group performance; NA, not applicable; NOS, not otherwise specified.

^a^ Results for 626 with 155 events; Cox regression with administrative censoring at 2 years; tumor size excluded from multivariate model given confounding with stage; pulmonary function test, lung age, mean estimated age, and normalized mean estimated age variables excluded from multivariate model given data available for only a subset of patients.

### Secondary End Points: Distant Metastasis and Pneumonitis

Overall 112 patients (17%) developed distant metastasis, with higher rates observed in those with more smoking pack-years, stage II vs I disease, or multifocal disease (eTable 3 in Supplement 1); 14 (2%) experienced symptomatic (grade ≥2) radiation pneumonitis, which was associated with higher BMI and stage II vs I disease (eTable 4 in Supplement 1). Neither face age nor lung age metrics were associated with an increased risk of distant metastasis or pneumonitis.

## Discussion

In this study of a large cohort of patients with early-stage NSCLC treated with definitive SBRT, we found that on average, patients’ biological age, when estimated by a deep learning-based model from facial photographs, was older than their chronological age. Face age was a significant biomarker associated with OS and early mortality, and extreme discordance between face age and chronological age was significantly associated with survival, with much older face age associated with worse OS and much younger face age associated with better OS. Moreover, patients with face age of 85 years or older had an increased risk for early mortality regardless of their chronological age. Such AI-based biomarkers may assist in guiding treatment decisions for this high-risk patient cohort with multiple medical comorbidities.

Patients with early-stage NSCLC who undergo definitive SBRT are often older and have substantial medical comorbidities.^7^ For instance, in a retrospective study by Grills et al^7^ comparing the outcomes of patients ineligible for anatomic lobectomy treated with wedge or segmentectomy vs SBRT, patients treated with SBRT were older and had more baseline medical comorbidities. Survival rates are poor, with a reported 3-year OS of 56% among patients treated with SBRT for inoperable disease, despite high local tumor control rates.^5^ Accordingly, assessment of such high-risk patients’ overall physiological health and life expectancy is important in making individualized treatment decisions.

While chronological age is reliably associated with survival among healthy patients, biological age offers a more accurate reflection of physiological health among unhealthy individuals, such as patients with cancer. While prior studies have investigated AI-assisted estimation of biological age using radiographic images (ie, chest, brain)^24,25,26,27^ and molecular data (ie, epigenomic, transcriptomic, proteomic, and metabolomic data),^28,29^ the use of a simple facial photograph to estimate the cumulative effects of time and biological changes presents a unique, accessible, and low-cost window to quantifying one’s biological age.^30,31^ Given the routine use of identification photographs in radiation oncology, integration of face age into clinical practice as a risk stratification tool would be easily feasible, with real-time computation of biological age estimates in the clinic. Combined with existing risk stratification tools and pertinent clinical information, such as ECOG PS, medical comorbidities, and geriatric evaluation, face age has the potential to refine life expectancy estimates and assist in tailoring personalized treatments. By identifying patients whose biological age diverges from their chronological age, clinicians could better balance treatment benefits against potential toxic effects. Our findings that patients with face ages of 85 years or older had significantly increased early mortality risk provides a clinically relevant threshold for identifying high-risk patients who might benefit from modified treatment approaches or active surveillance.

We also tested the interaction of face age with a complementary measure derived from spirometry. Lung age was only weakly correlated with both chronological age and face age, suggesting it captures distinct physiological domains. In this high-risk lung cancer population, in which smoking-related pulmonary compromise is prevalent, face age remained independently associated with survival in multivariate models adjusting for lung age, confirming they capture complementary physiological domains.

## Limitations

Limitations of this study should be considered when interpreting the results. The generalizability of the face age model is predominantly limited to patients aged 60 years or older given the training and validation for the model were conducted with a focus on this age range. However, most patients diagnosed with NSCLC are aged 65 years and older and within the appropriate age range for optimal model performance. While the face age model was developed using ethnically diverse datasets with approximately 45% White individuals, the patient cohort in this study was predominantly White. All patients were also treated in a single state at clinic sites affiliated with a single large cancer center, which may reflect different demographic characteristics and clinical practices (ie, referral pattern for SBRT) compared with other regions within and outside of the United States. Accordingly, differential performance across racial and ethnic groups cannot be excluded based on our data, and further evaluation of the model in larger and more diverse patient populations is necessary before broader clinical implementation. External influences that could affect the facial aging process, such as cosmetic procedures and sun exposure, were not factored into our study. Future studies are needed to understand the impact of such external factors and explore methods for adjusting the model accordingly. Moreover, while poor baseline PFTs (FEV_1_, Dlco) and older lung age based on FEV_1_ are risk factors for worse clinical outcomes, including survival,^32,33,34,35^ only a subset of patients had available PFT data prior to SBRT with tests obtained at varying time points. Further assessment of face age with the incorporation of PFT data is necessary to establish whether face age is a biomarker independent of other existing factors for clinical outcomes. Additionally, toxic effects data were limited to symptomatic radiation pneumonitis, and other relevant end points, such as fatigue and chest wall pain, were not systematically collected. Lastly, while we did not observe significant associations between face age and secondary end points, including distant metastasis and symptomatic pneumonitis, more studies in larger datasets are needed to evaluate whether advanced biological age as measured by face age is associated with cancer outcomes and cancer therapy toxic effects.

Rigorous training and validation of the face age model in larger, prospective settings to address its current limitations are imperative prior to real-world implementation. Moreover, ethical concerns surrounding personal health information regulation (ie, data storage and sharing), informed consent on the use and implications of facial photographs, and potential misuse of the model by entities such as life insurance and product marketing must be addressed, although clinical implementation would utilize photographs already routinely obtained for patient identification in radiation oncology.

## Conclusions

In this study, face age was associated with OS and early mortality, and chronological age was not. In particular, patients with a face age of 85 years or older had a significantly increased risk for early mortality. These findings suggest that deep learning-based quantification of biological age from facial images may be an effective biomarker for overall physiological health and early mortality among patients with early-stage NSCLC undergoing SBRT. Lung age captured a distinct physiological domain independent of face age (*r* = 0.07), and face age remained significant in multivariate models adjusting for lung age, suggesting complementary value for risk stratification in this high-risk population.

## Notes

Supplement 1. **eTable 1**. Sensitivity Analysis—Results for Face Age Variables in Multivariate Cox Regression for Overall Survival With Incorporation of Dlco and Lung Age Variables Available in a Subset of Patients **eTable 2**. Sensitivity Analysis—Cox Regression for 2-Year Mortality With Dlco and Lung Age **eTable 3. **Univariate and Multivariate Cox Regression for Distant Metastasis **eTable 4. **Univariate and Multivariate Logistic Regression for Symptomatic Radiation Pneumonitis **eTable 5.** Univarirate Analysis for Overall Survival and 2-Year Mortality **eFigure 1.** CONSORT Diagram of Patient Selection **eFigure 2.** Associations and Distributions of Age-Related Metrics in Patients With Lung Cancer

Supplement 2.
Data Sharing Statement
