# Supplementary material for: Multimodal Assessment of Biological Age Following Radiation Therapy Among Patients With Early-Stage NSCLC
Source: JAMA Netw Open. 2026 Apr 8;9(4):e264872. doi: 10.1001/jamanetworkopen.2026.4872 (PMC13063086; doi:10.1001/jamanetworkopen.2026.4872)
Supplement: Supplement 1. — eTable 1. Sensitivity Analysis—Results for Face Age Variables in Multivariate Cox Regression for Overall Survival With Incorporation of Dlco and Lung Age Variables Available in a Subset of Patients eTable 2. Sensitivity Analysis—Cox Regression for 2-Year Mortality With Dlco and Lung Age eTable 3. Univariate and Multivariate Cox Regression for Distant Metastasis eTable 4. Univariate and Multivariate Logistic Regression for Symptomatic Radiation Pneumonitis eTable 5. Univarirate Analysis for Overall Survival and 2-Year Mortality eFigure 1. CONSORT Diagram of Patient Selection eFigure 2. Associations and Distributions of Age-Related Metrics in Patients With Lung Cancer [file jamanetwopen-e264872-s001.pdf]

Supplemental Online Content

Lee G, Haugg F, Bontempi D, et al. Multimodal assessment of biological age following radiation therapy among patients with early-stage NSCLC. *JAMA Netw Open.* 2026;9(4):e264872. doi:10.1001/jamanetworkopen.2026.4872

eTable 1. Sensitivity Analysis—Results for Face Age Variables in Multivariate Cox Regression for Overall Survival With Incorporation of DLCO and Lung Age Variables Available in a Subset of Patients

eTable 2. Sensitivity Analysis—Cox Regression for 2-Year Mortality With DLCO and Lung Age

eTable 3. Univariate and Multivariate Cox Regression for Distant Metastasis

eTable 4. Univariate and Multivariate Logistic Regression for Symptomatic Radiation Pneumonitis

eTable 5. Univariate Analysis for Overall Survival and 2-Year Mortality

eFigure 1. CONSORT Diagram of Patient Selection

eFigure 2. Associations and Distributions of Age-Related Metrics in Patients With Lung Cancer

This supplemental material has been provided by the authors to give readers additional information about their work.

© 2026 Lee G et al. *JAMA Network Open*.

eTable 1. Sensitivity Analysis—Results for Face Age Variables in Multivariate Cox Regression for Overall Survival With Incorporation of DLCO and Lung Age Variables Available in a Subset of Patients

**Cox regression**	**Multivariate model[a] (DLCO)**		**Multivariate model[b] (Lung age)**		**Multivariate model[c] (DLCO and lung age)**	
**Face age Variables**	HR (95% CI)	P value	HR (95% CI)	P value	HR (95% CI)	P value
Face age (decade)	1.27 (0.96-1.69)	0.09	1.38 (1.09-1.75)	0.007	1.28 (0.97-1.71)	0.085
Face age ≥85 years	1.26 (0.83-1.91)	0.281	1.49 (1.05-2.11)	0.026	1.31 (0.86-2.00)	0.207
Face age–age (decade)	1.27 (0.96-1.69)	0.09	1.38 (1.09-1.75)	0.007	1.28 (0.97-1.71)	0.085
Face age–age ≥10 years	1.30 (0.80-2.11)	0.287	1.31 (0.85-2.01)	0.217	1.27 (0.78-2.07)	0.336
Face age–age ≤-5 years	0.56 (0.32-1.00)	0.048	0.56 (0.35-0.90)	0.018	0.56 (0.31-1.00)	0.052

[a]n=339 with 155 events; adjusted for age, sex, ECOG PS, smoking pack years, stage, histology, DLCO (<40% vs ≥40%)

[b]n=449 with 215 events; adjusted for age, sex, ECOG PS, smoking pack years, stage, histology, Lung age

[c]n=331 with 153 events; adjusted for age, sex, ECOG PS, smoking pack years, stage, histology, DLCO (<40% vs ≥40%), Lung age

Abbreviations: DLCO, diffusing capacity for carbon monoxide; ECOG PS, Eastern Cooperative Oncology Group performance status

© 2026 Lee G et al. *JAMA Network Open*.

eTable 2. Sensitivity Analysis—Cox Regression for 2-Year Mortality With DLCO and Lung Age

**Cox regression**	**Multivariate model[a] (DLCO)**		**Multivariate model[b] (Lung age)**		**Multivariate model[c] (DLCO and lung age)**	
**Face age Variables**	HR (95% CI)	P value	HR (95% CI)	P value	HR (95% CI)	P value
Face age (decade)	1.14 (0.77-1.67)	0.516	1.30 (0.95-1.77)	0.102	1.16 (0.79-1.71)	0.458
Face age ≥85 years	1.36 (0.76-2.42)	0.3	1.51 (0.93-2.42)	0.093	1.33 (0.74-2.39)	0.339
Face age–age (decade)	1.14 (0.77-1.67)	0.516	1.30 (0.95-1.77)	0.102	1.16 (0.79-1.71)	0.458
Face age–age ≥10 years	1.46 (0.78-2.74)	0.242	1.60 (0.92-2.78)	0.093	1.50 (0.80-2.83)	0.209
Face age–age ≤-5 years	0.59 (0.26-1.32)	0.201	0.60 (0.31-1.13)	0.114	0.62 (0.28-1.38)	0.238

[a]n=339 with 81 events; adjusted for age, sex, ECOG PS, smoking pack years, stage, histology, DLCO (<40% vs ≥40%)

[b]n=449 with 116 events; adjusted for age, sex, ECOG PS, smoking pack years, stage, histology, Lung age

[c]n=331 with 80 events; adjusted for age, sex, ECOG PS, smoking pack years, stage, histology, DLCO (<40% vs ≥40%), Lung age

Abbreviations: DLCO, diffusing capacity for carbon monoxide; ECOG PS, Eastern Cooperative Oncology Group performance status

© 2026 Lee G et al. *JAMA Network Open*.

eTable 3. Univariate and Multivariate Cox Regression for Distant Metastasis

**Cox regression**	**Univariate[a]**		**Multivariate model[b] (Face age)**		**Multivariate model[b] (Face age ≥85 years)**		**Multivariate model[b] (Face age–age)**		**Multivariate model[b] (Face age–age ≥10 years)**		**Multivariate model[b] (Face age–age ≤-5 years)**	
Variables	HR (95% CI)	P value	HR (95% CI)	P value	HR (95% CI)	P value	HR (95% CI)	P value	HR (95% CI)	P value	HR (95% CI)	P value
Age (decade)	0.83 (0.65-1.06)	0.131	0.79 (0.57-1.10)	0.161	0.90 (0.67-1.20)	0.465	0.99 (0.74-1.32)	0.929	0.93 (0.71-1.22)	0.598	0.94 (0.72-1.23)	0.657
Face age	--	--	--	--	--	--	--	--	--	--	--	--
Face age (decade)	0.98 (0.77-1.25)	0.876	1.25 (0.91-1.71)	0.174	--	--	--	--	--	--	--	--
Face age ≥85 years	0.88 (0.55-1.41)	0.59	--	--	1.04 (0.61-1.79)	0.878	--	--	--	--	--	--
Face age-age (decade)	1.24 (0.94-1.63)	0.13	--	--	--	--	1.25 (0.91-1.71)	0.174	--	--	--	--
Face age-age ≥10 years	1.35 (0.80-2.26)	0.259	--	--	--	--	--	--	1.23 (0.71-2.14)	0.464	--	--
Face age-age ≤-5 years	0.75 (0.42-1.34)	0.33	--	--	--	--	--	--	--	--	0.69 (0.35-1.34)	0.27
Sex, male vs female	1.24 (0.85-1.81)	0.266	--	--	--	--	--	--	--	--	--	--
Race/ethnicity[c]	--	--	--	--	--	--	--	--	--	--	--	--
White	Ref	--	--	--	--	--	--	--	--	--	--	--
Black	1.52 (0.62-3.73)	0.361	--	--	--	--	--	--	--	--	--	--
Asian	1.63 (0.23-11.69)	0.627	--	--	--	--	--	--	--	--	--	--
Other	0.00 (0.00-Inf)	0.995	--	--	--	--	--	--	--	--	--	--
ECOG PS	--	--	--	--	--	--	--	--	--	--	--	--
0-1	Ref	--	--	--	--	--	--	--	--	--	--	--
2-4	0.95 (0.62-1.46)	0.807	--	--	--	--	--	--	--	--	--	--
Smoking	--	--	--	--	--	--	--	--	--	--	--	--
Never	Ref	--	--	--	--	--	--	--	--	--	--	--
Former	2.24 (0.77-6.57)	0.14	--	--	--	--	--	--	--	--	--	--
Active	1.95 (0.72-5.32)	0.191	--	--	--	--	--	--	--	--	--	--
Smoking pack years	1.01 (1.00-1.01)	0.018	1.01 (1.00-1.01)	0.021	1.01 (1.00-1.01)	0.02	1.01 (1.00-1.01)	0.021	1.01 (1.00-1.01)	0.019	1.01 (1.00-1.01)	0.022
Stage	--	--	--	--	--	--	--	--	--	--	--	--
I (IA1-IB)	Ref	--	Ref	--	Ref	--	Ref	--	Ref	--	Ref	--
II (IIA-IIB)	3.04 (1.33-6.95)	0.008	3.28 (1.41-7.62)	0.006	3.08 (1.33-7.14)	0.009	3.28 (1.41-7.62)	0.006	3.09 (1.34-7.14)	0.008	3.10 (1.34-7.16)	0.008
Multifocal	1.66 (1.14-2.40)	0.008	1.75 (1.18-2.58)	0.005	1.76 (1.19-2.61)	0.004	1.75 (1.18-2.58)	0.005	1.77 (1.20-2.62)	0.004	1.75 (1.19-2.58)	0.005
Histology	--	--	--	--	--	--	--	--	--	--	--	--
Adenocarcinoma	Ref	--	--	--	--	--	--	--	--	--	--	--
Squamous cell carcinoma	1.03 (0.58-1.81)	0.93	--	--	--	--	--	--	--	--	--	--
Other/NOS	0.85 (0.36-2.01)	0.717	--	--	--	--	--	--	--	--	--	--
Unknown (not biopsied)	0.73 (0.48-1.11)	0.136	--	--	--	--	--	--	--	--	--	--
Tumor size (cm)	1.23 (1.01-1.50)	0.037	--	--	--	--	--	--	--	--	--	--
BMI	0.99 (0.96-1.02)	0.589	--	--	--	--	--	--	--	--	--	--
FEV1 (L)	1.03 (0.73-1.45)	0.855	--	--	--	--	--	--	--	--	--	--
FEV1 % predicted	1.00 (0.99-1.01)	0.681	--	--	--	--	--	--	--	--	--	--
DLCO	1.00 (0.94-1.07)	0.929	--	--	--	--	--	--	--	--	--	--
DLCO % predicted	1.00 (0.99-1.01)	0.907	--	--	--	--	--	--	--	--	--	--
DLCO % predicted <40%	0.86 (0.48-1.55)	0.621	--	--	--	--	--	--	--	--	--	--
Lung age (decade)	1.03 (0.93-1.15)	0.556	--	--	--	--	--	--	--	--	--	--

[a]n=670 with 112 events

[b]n=626 with 106 events; adjusted for age, smoking pack years, stage, multifocal disease; tumor size was excluded from the multivariate model due to confounding with stage

[c]Race/ethnicity was self-reported

Abbreviations: ECOG PS, Eastern Cooperative Oncology Group performance status; NOS, not otherwise specified; BMI, body mass index; FEV1, forced expiratory volume in 1 second; DLCO, diffusing capacity for carbon monoxide.

© 2026 Lee G et al. *JAMA Network Open*.

eTable 4. Univariate and Multivariate Logistic Regression for Symptomatic Radiation Pneumonitis

**Logistic regression**	**Univariate**[a]		**Multivariate model**[b] **(Face age)**		**Multivariate model**[b] **(Face age ≥85 years)**		**Multivariate model**[b] **(Face age–age)**		**Multivariate model**[b] **(Face age–age ≥10 years)**		**Multivariate model**[b] **(Face age–age ≤-5 years)**	
Variables	OR (95% CI)	P value	OR (95% CI)	P value	OR (95% CI)	P-value	OR (95% CI)	P value	OR (95% CI)	P value	OR (95% CI)	P value
Age (decade)	0.72 (0.36-1.43)	0.343	0.91 (0.38-2.19)	0.834	0.89 (0.42-1.91)	0.764	0.63 (0.29-1.38)	0.248	0.62 (0.29-1.32)	0.211	0.74 (0.34-1.60)	0.443
Face age	--	--	--	--	--	--	--	--	--	--	--	--
Face age (decade)	0.56 (0.30-1.04)	0.066	0.69 (0.31-1.56)	0.372	--	--	--	--	--	--	--	--
Face age ≥85 years	<0.01 (<0.01->99.99)	0.991	--	--	<0.01 (<0.01->99.99)	0.991	--	--	--	--	--	--
Face age-age (decade)	0.69 (0.33-1.45)	0.324	--	--	--	--	0.69 (0.31-1.56)	0.372	--	--	--	--
Face age-age ≥10 years	<0.01 (<0.01->99.99)	0.989	--	--	--	--	--	--	<0.01 (<0.01->99.99)	0.989	--	--
Face age-age ≤-5 years	0.97 (0.21-4.41)	0.971	--	--	--	--	--	--	--	--	0.90 (0.18-4.47)	0.894
Sex, male vs female	2.08 (0.71-6.07)	0.179	--	--	--	--	--	--	--	--	--	--
Race/ethnicity[c]	--	--	--	--	--	--	--	--	--	--	--	--
White	Ref	--	--	--	--	--	--	--	--	--	--	--
Black	<0.01 (<0.01->99.99)	0.992	--	--	--	--	--	--	--	--	--	--
Asian	<0.01 (<0.01->99.99)	0.996	--	--	--	--	--	--	--	--	--	--
Other	<0.01 (<0.01->99.99)	0.996	--	--	--	--	--	--	--	--	--	--
ECOG PS	--	--	--	--	--	--	--	--	--	--	--	--
0-1	Ref	--	--	--	--	--	--	--	--	--	--	--
2-4	0.64 (0.18-2.30)	0.49	--	--	--	--	--	--	--	--	--	--
Smoking	--	--	--	--	--	--	--	--	--	--	--	--
Never	Ref	--	--	--	--	--	--	--	--	--	--	--
Former	<0.01 (<0.01->99.99)	0.988	--	--	--	--	--	--	--	--	--	--
Active	1.04 (0.13-8.14)	0.971	--	--	--	--	--	--	--	--	--	--
Smoking pack years	0.98 (0.96-1.00)	0.108	--	--	--	--	--	--	--	--	--	--
Stage	--	--	--	--	--	--	--	--	--	--	--	--
I (IA1-IB)	Ref	--	Ref	--	Ref	--	Ref	--	Ref	--	Ref	--
II (IIA-IIB)	6.26 (1.30-30.19)	0.022	4.84 (0.96-24.51)	0.056	4.41 (0.87-22.43)	0.074	4.84 (0.96-24.51)	0.056	4.92 (0.96-25.24)	0.056	4.89 (0.97-24.81)	0.055
Multifocal	1.40 (0.49-4.05)	0.531	--	--	--	--	--	--	--	--	--	--
Histology	--	--	--	--	--	--	--	--	--	--	--	--
Adenocarcinoma	Ref	--	--	--	--	--	--	--	--	--	--	--
Squamous cell carcinoma	2.09 (0.51-8.56)	0.303	--	--	--	--	--	--	--	--	--	--
Other/NOS	<0.01 (<0.01->99.99)	0.99	--	--	--	--	--	--	--	--	--	--
Unknown (not biopsied)	0.90 (0.25-3.23)	0.87	--	--	--	--	--	--	--	--	--	--
Tumor size (cm)	1.62 (1.01-2.59)	0.045	--	--	--	--	--	--	--	--	--	--
BMI	1.11 (1.03-1.19)	0.004	1.09 (1.01-1.18)	0.019	1.09 (1.01-1.17)	0.021	1.09 (1.01-1.18)	0.019	1.09 (1.02-1.17)	0.016	1.10 (1.02-1.18)	0.009
FEV1 (L)	1.70 (0.77-3.76)	0.187	--	--	--	--	--	--	--	--	--	--
FEV1 % predicted	1.00 (0.98-1.02)	0.908	--	--	--	--	--	--	--	--	--	--
DLCO	1.01 (0.87-1.17)	0.935	--	--	--	--	--	--	--	--	--	--
DLCO % predicted	0.99 (0.96-1.02)	0.569	--	--	--	--	--	--	--	--	--	--
DLCO % predicted <40%	1.19 (0.35-4.03)	0.784	--	--	--	--	--	--	--	--	--	--
Lung age (decade)	0.98 (0.74-1.30)	0.902	--	--	--	--	--	--	--	--	--	--

[a]n=670 with 14 events

[b]n=666 with 14 events; adjusted for age, stage, BMI; tumor size was excluded from the multivariate model due to confounding with stage

[c]Race/ethnicity was self-reported

Abbreviations: ECOG PS, Eastern Cooperative Oncology Group performance status; NOS, not otherwise specified; BMI, body mass index; FEV1, forced expiratory volume in 1 second; DLCO, diffusing capacity for carbon monoxide.

© 2026 Lee G et al. *JAMA Network Open*.

eTable 5. Univariate Analysis for Overall Survival and 2-Year Mortality

Variable	Univariate[a]	
	HR (95% CI)	P value
**Overall survival**		
Chronological age (decade)	1.26 (1.08-1.47)	.003
Face age		
Face age (decade)	1.40 (1.20-1.64)	<.001
Face age ≥85 y	1.46 (1.13-1.89)	.004
Face age − age (decade)	1.12 (0.95-1.32)	.19
Face age − age ≥10 y	1.12 (0.80-1.57)	.51
Face age − age ≤−5 y	0.71 (0.49-1.03)	.07
Sex, male vs female	1.55 (1.23-1.96)	<.001
Race and ethnicity[b]		
White	1 [Reference]	NA
Black	0.94 (0.47-1.90)	.87
Asian	0.74 (0.18-2.99)	.67
Other	0.70 (0.10-5.00)	.72
ECOG PS		
0-1	1 [Reference]	NA
2-4	2.74 (2.17-3.45)	<.001
Smoking		
Never	1 [Reference]	NA
Former	1.26 (0.71-2.24)	.42
Active	1.31 (0.79-2.18)	.29
Smoking pack-years	1.00 (1.00-1.01)	.04
Stage		
I (IA1-IB)	1 [Reference]	NA
II (IIA-IIB)	2.36 (1.32-4.22)	.004
Multifocal	0.94 (0.74-1.19)	.60
Histology		
Adenocarcinoma	1 [Reference]	NA
Squamous cell carcinoma	1.87 (1.35-2.61)	<.001
Other or NOS	1.46 (0.92-2.34)	.11
Unknown (not biopsied)	0.95 (0.72-1.25)	.73
Tumor size, cm	1.18 (1.05-1.34)	.006
BMI	0.99 (0.97-1.01)	.24
FEV1, L	0.95 (0.77-1.18)	.65
FEV1, % predicted	1.00 (0.99-1.00)	.17
Dlco	0.93 (0.89-0.97)	<.001
Dlco, % predicted	0.98 (0.97-0.99)	<.001
Dlco, % predicted <40%	1.71 (1.24-2.35)	.001
Lung age (decade)	1.11 (1.03-1.18)	.004
Mean estimated age (decade)	1.30 (1.14-1.48)	<.001
Normalized mean estimated age	1.59 (1.31-1.94)	<.001
**2-y Mortality**		
Chronological age (decade)	1.13 (0.93-1.39)	.22
Face age		
Face age (decade)	1.29 (1.04-1.59)	.02
Face age ≥85 y	1.39 (0.98-1.96)	.06
Face age − age (decade)	1.16 (0.92-1.45)	.21
Face age − age≥10 y	1.29 (0.84-1.99)	.25
Face age − age ≤-5 y	0.71 (0.43-1.15)	.16
Sex, male vs female	1.61 (1.18-2.19)	.003
Race and ethnicityb		
White	1 [Reference]	NA
Black	1.03 (0.42-2.52)	.94
Asian	2.32 (0.57-9.35)	.24
Additional groups	0.00 (0.00-∞)	.99
ECOG PS		
0-1	1 [Reference]	NA
2-4	3.19 (2.34-4.35)	<.001
Smoking		
Never	1 [Reference]	NA
Former	1.29 (0.55-3.03)	.56

© 2026 Lee G et al. *JAMA Network Open*.

Active	1.60 (0.75-3.43)	.22
Smoking pack years	1.00 (1.00-1.01)	.03
Stage		
I (IA1-IB)	1 [Reference]	NA
II (IIA-IIB)	2.95 (1.56-5.61)	<.001
Multifocal	0.88 (0.64-1.21)	.43
Histology		
Adenocarcinoma	1 [Reference]	NA
Squamous cell carcinoma	1.98 (1.28-3.08)	.002
Other or NOS	1.01 (0.45-2.25)	.98
Unknown (not biopsied)	1.05 (0.72-1.53)	.81
Tumor size, cm	1.27 (1.09-1.48)	.003
BMI	0.99 (0.97-1.02)	.65
FEV1, L	0.98 (0.74-1.31)	.91
FEV1, % predicted	1.00 (0.99-1.00)	.34
Dlco	0.91 (0.85-0.96)	<.001
Dlco, % predicted	0.97 (0.96-0.99)	<.001
Dlco, % predicted <40%	2.06 (1.33-3.17)	.001
Lung age (decade)	1.10 (1.00-1.21)	.045
Mean estimated age (decade)	1.27 (1.07-1.51)	.007
Normalized mean estimated age	1.50 (1.15-1.94)	.002

Abbreviations: BMI, body mass index (calculated as weight in kilograms divided by height in meters squared); DLCO, diffusing capacity for carbon monoxide; FEV1, forced expiratory volume in 1 second.

[a]670 participants with 299 events for overall survival outcome; 670 with 161 events within 2 years for 2-year mortality outcome.

[b]Race and ethnicity were self-reported.

© 2026 Lee G et al. *JAMA Network Open*.

eFigure 1. CONSORT Diagram of Patient Selection

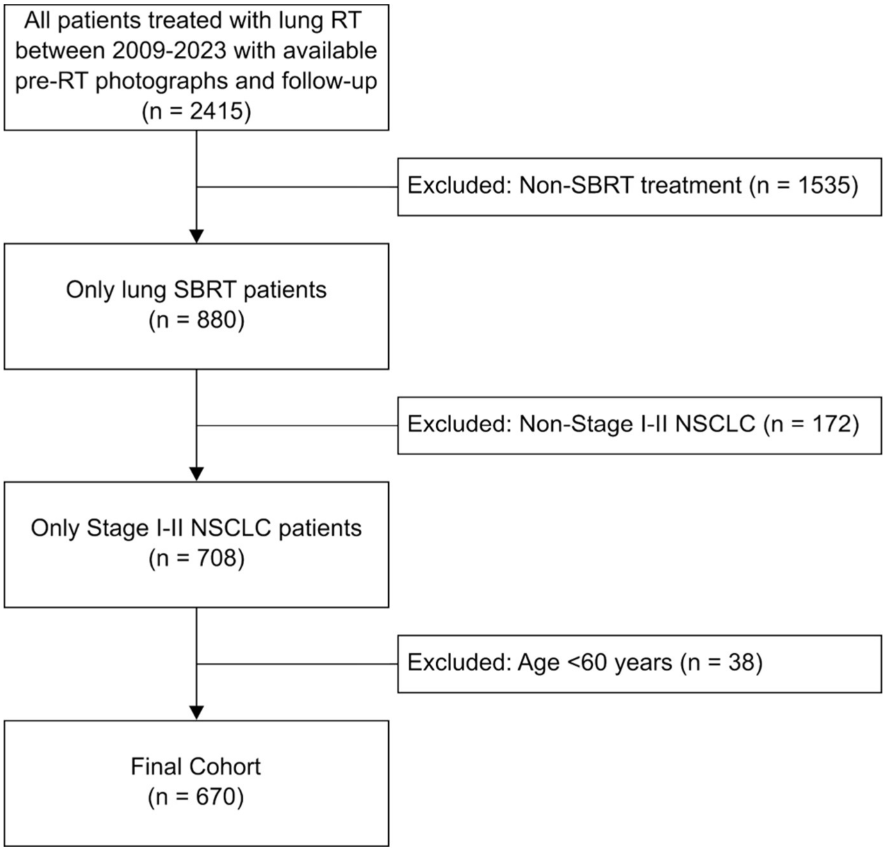

This retrospective cohort study was approved by the institutional review board; a total of 670 patients aged ≥60 years who underwent definitive stereotactic body radiotherapy (SBRT) for early-stage (AJCC 8th edition T1–3N0M0, stage I-II) non-small cell lung cancer (NSCLC) with or without biopsy confirmation between 2009 and 2023 at six clinics were identified. Additionally, to be included in the study, patients also had to have a pre-SBRT facial photograph used for radiation therapy identification and available follow-up data. RT= Radiation Therapy; SBRT = Stereotactic Body Radiation Therapy; NSCLC: Non-Small Cell Lung Cancer.

© 2026 Lee G et al. *JAMA Network Open*.

eFigure 2. Associations and Distributions of Age-Related Metrics in Patients With Lung Cancer

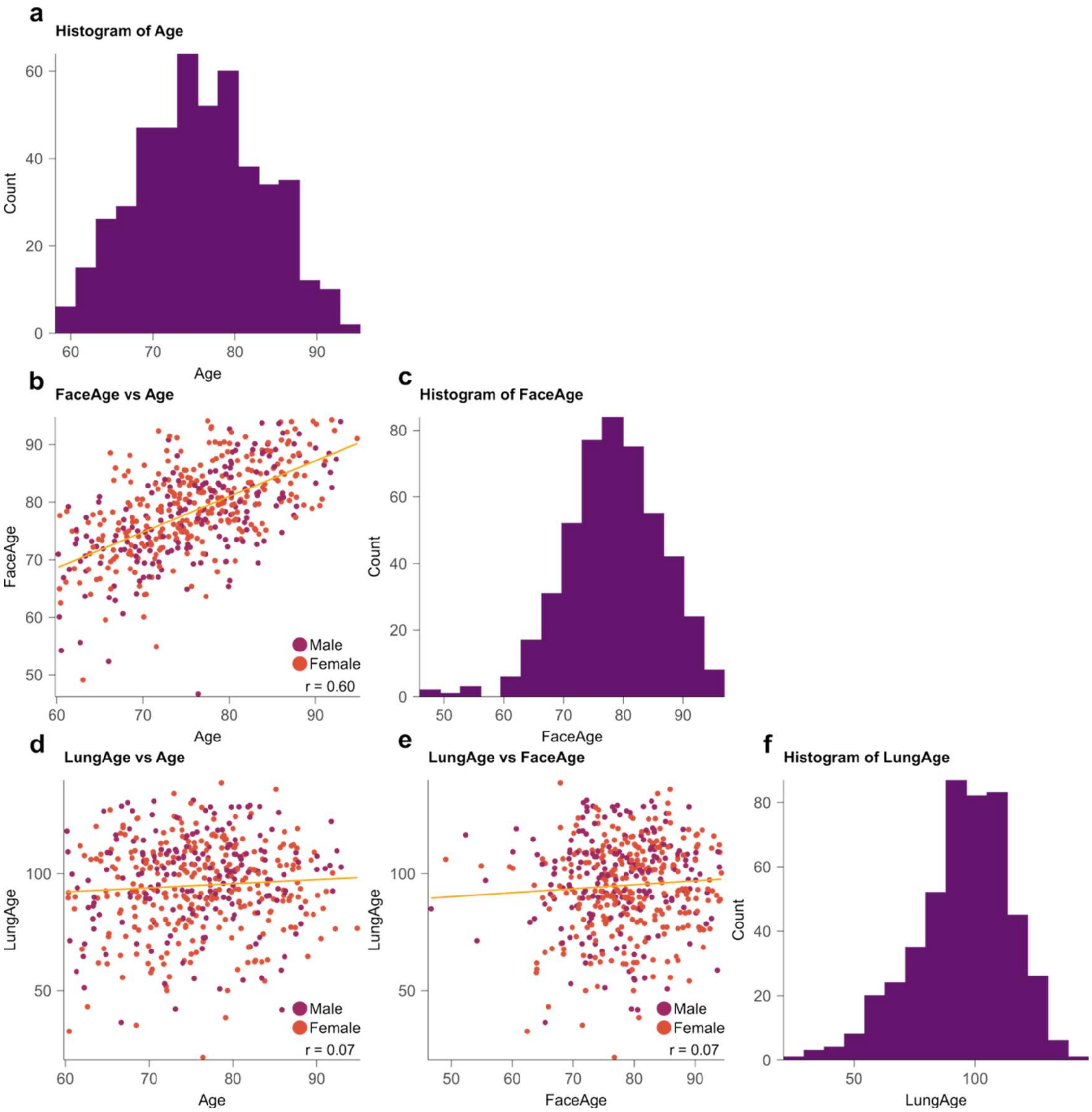

This figure illustrates the relationships between chronological age, Face age, and Lung age, as well as their individual distributions in our cohort of lung cancer patients aged 60 and above.

a, Histogram of chronological age.

b, Scatter plot of Face age vs. chronological age. There is a moderate positive correlation (r=0.60) between Face age and chronological age. The orange line represents the linear regression fit. Red points indicate female patients, while purple points represent male patients.

c, Histogram of Face age.

d, Scatter plot of Lung age vs. chronological age. There is a very weak correlation (r=0.07) between Lung age and chronological age.

e, Scatter plot of Lung age vs. Face age. There is a very weak correlation (r=0.07) between Lung age and Face age.

f, Histogram of Lung age.

© 2026 Lee G et al. *JAMA Network Open*.
