# Supplementary material for: Multimodal Assessment of Biological Age Following Radiation Therapy Among Patients With Early-Stage NSCLC
Source: JAMA Netw Open. 2026 Apr 8;9(4):e264872. doi: 10.1001/jamanetworkopen.2026.4872 (PMC13063086; doi:10.1001/jamanetworkopen.2026.4872)
Supplement: Supplement 2. — Data Sharing Statement [file jamanetwopen-e264872-s002.pdf]

# Data Sharing Statement

Lee. Multimodal Assessment of Biological Age Following Radiation Therapy Among Patients With Early-Stage NSCLC. *JAMA Netw Open*. Published April 08, 2026. doi:10.1001/jamanetworkopen.2026.4872

**Data**
**Data available:** No

**Additional Information**
**Explanation for why data not available:** Individual participant data cannot be shared publicly due to institutional privacy policies and the inclusion of facial images.
